# The Effects of Davallic Acid from *Davallia divaricata* Blume on Apoptosis Induction in A549 Lung Cancer Cells

**DOI:** 10.3390/molecules171112938

**Published:** 2012-11-01

**Authors:** An-Sheng Cheng, We-Chang Chang, Yu-Hsiang Cheng, Kai-Yu Chen, Kai-Hsien Chen, Tsu-Liang Chang

**Affiliations:** 1 Department of Horticulture and Landscape Architecture, National Taiwan University, 1 Roosevelt Road Section 4, Taipei 10617, Taiwan; Email: d99628009@ntu.edu.tw (A.-S.C.); kaic@sinetics.com.tw (K.-H.C.); 2 Institute of Food Science and Technology, National Taiwan University, 59 Roosevelt Road Section 4, Taipei 10617, Taiwan; Email: d99641001@ntu.edu.tw; 3 Department of Science, University of Auckland, 23 Symonds Street, Auckland 1142, New Zealand; Email: yche556@aucklanduni.ac.nz; 4 Department of Life Sciences, National Cheng Kung University, 1 University Road, Tainan 70101, Taiwan; Email: C5497105@email.ncku.edu.tw

**Keywords:** *Davallia divaricata* Blume, davallic acid, lung cancer, apoptotic activity

## Abstract

Traditional or folk medicinal herbs continue to be prescribed in the treatment of various diseases and conditions in many cultures. Recent scientific efforts have focused on the potential roles of extracts of traditional herbs as alternative and complementary medications for cancer treatment. In Taiwan, *Davallia divaricata* Blume has been traditionally employed in folk medicine for therapy of lung cancer, davallic acid being the major active compound of *D. divaricata* Blume. In this study, we investigated the inhibitory activity of davallic acid on the proliferation of A549 lung cancer cells. Davallic acid was extracted from *D. divaricata* Blume, and its effects on cell viability, cell cycle distribution, ROS level, and apoptotic protein expression in A549 cells were determined. Davallic acid significantly induced reactive oxygen species (ROS) generation as well as caspase-3, -8, and -9 activation, thereby repressing A549 cell growth and elevating apoptotic activity. Since lung cancer has a high incidence of recurrence, these results indicate that davallic acid may have the potential to be a natural anti-lung cancer compound, and may provide a basis for further study of its use in combating cancer.

## 1. Introduction

Lung cancer is the cause of approximately 20% of tumor-related deaths worldwide; in Taiwan, it is the first- and the second-most lethal cancer type for females and males, respectively. Approximately 70% of lung cancer patients die from metastasis. Cancer metastasis refers to the spread of cancer cells from the primary neoplasm to distant sites and the growth of secondary tumors at sites distant from the primary tumor [1].

*Davallia divaricata* Blume is a fern perennial (genus *Davallia*, family Davalliaceae). It is a folk remedy “Gusuibu” known for its use in traditional Chinese medicine [2]. The dry rhizomes display many biological applications with therapeutic effects against diseases such as musculoskeletal traumatic disorders [3], rheumatoid arthritis and osteoporosis [4]. Chemical examination of this plant have resulted in the isolation of a series of compounds, including davallic acid [5,6], (+)-catechin-3-*O*-β-D-allopyranoside, (−)epicatechin-3-*O*-β-D-allopyranoside, procyanidins B-1 and B-2, trimeric procyanidin [7], β-carboxymethyl-(−)-epicatechin, and epiafzelechin-(4β→6)-epicatechin-(4β→8)-epicatechin-(4β→6)-epicatechin [8]. The structure of davallic acid was determined in 1965 [6]. Since then, phytochemical studies have been conducted on the active constituents of these rhizomes however analytical tests of the activities of davallic acid have not been reported.

Traditional or folk medicinal herbs continue to be prescribed in the treatment of various diseases and conditions in many cultures. In Taiwan, *D. divaricata* Blume has been employed traditionally for the therapy of lung cancer in folk medicine although its mechanism remains unknown; moreover, its efficacy in combating cancer also remains unverified. Davallic acid was identified as a major active compound of *D. divaricata* Blume and its structure was certified by Lin *et al.* [6]. In the present study, we evaluated the capacity of davallic acid to inhibit lung cancer cell growth. Using large amounts of davallic acid purified from *D. divaricata* Blume, we examined the bioactivity of davallic acid by investigating the anti-lung cancer efficacy of davallic acid and it mechanism of action.

Some phytochemicals arrest the cell cycle in cancer cells by altering signal transduction pathways and *via* the induction of apoptosis through the generation of reactive oxygen species (ROS); ensuing cell death is accompanied by the activation of certain stress kinases [9]. Different caspases mediate the two apoptotic signaling pathways. Initiation of the Fas signaling pathway by either Fas/death receptors or tumor necrosis factor (TNF)-receptor causes recruitment of Fas-associated protein with death domain (FADD) via interactions between the death domains of Fas and FADD. Caspase-8 is activated by the binding of procaspase-8 to the Fas/FADD complex, in turn activating caspase-3 and inducing apoptosis. The second apoptotic pathway is regulated by the mitochondrial release of cytochrome *c*, resulting in the activation of caspase-9 and caspase-3 [10].

## 2. Results

### 2.1. Structure Elucidation of Davallic Acid

**Figure 1:**
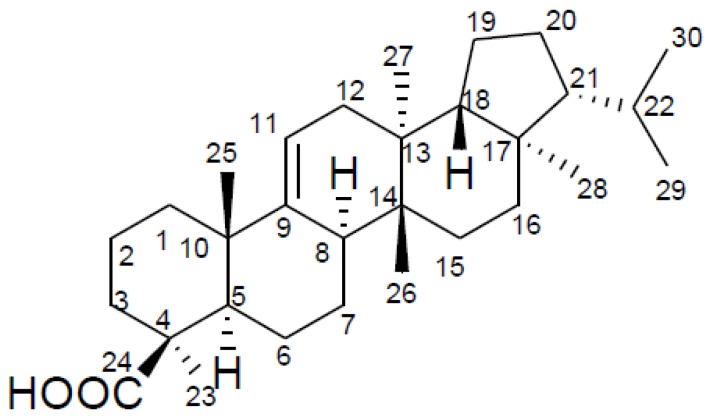
The structure of davallic acid.

The EtOH extracts of dry *D. divaricata* Blume rhizomes were successively partitioned which resulted in isolation of a crystalline white powder, m.p. 283–284 °C; 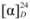 : +3.6° (*c* = 0.87, CHCl_3_). The LC-MS/MS analysis revealed a major peak at *m/z* 439.3 (M−H^−^) with very little fragmentation that confirmed the molecular formula to be C_30_H_48_O_2_ (MW 440). Further MS/MS analysis yielded very little fragment ions, which agrees with the triterpene compound nature. The compound was therefore identified as davallic acid (Figure 1) [11] based on its ^1^H- and ^13^C-NMR spectrum (Table 1) along with data comparisons with literature data.

**Table 1:** ^1^H and ^13^C-NMR spectroscopic data for davallic acid.

Position	*δ*_H_ (*J* in Hz)	*δ*_C_
1		41.8
2		19.5
3		42.7
4		35.8
5		46.9
6		18.2
7		19.8
8		39.4
9		149.7
10		38.0
11	5.36 (1H, m)	116.7
12		36.5
13		37.5
14		38.3
15		28.1
16		36.4
17		44.3
18		51.7
19		20.2
20		28.0
21		59.4
22		29.0
23	1.25 (3H, s)	30.5
24		183.9
25	1.02 (3H, s)	23.1
26	0.75 (3H, s)	15.4
27	0.83 (3H, s)	15.3
28	0.78 (3H, s)	13.7
29	0.85 (3H, d, *J* = 6.7)	21.9
30	0.90 (3H, d, *J* = 6.7)	22.7

### 2.2. The Effect of Davallic Acid on A549 Viability

**Figure 2:**
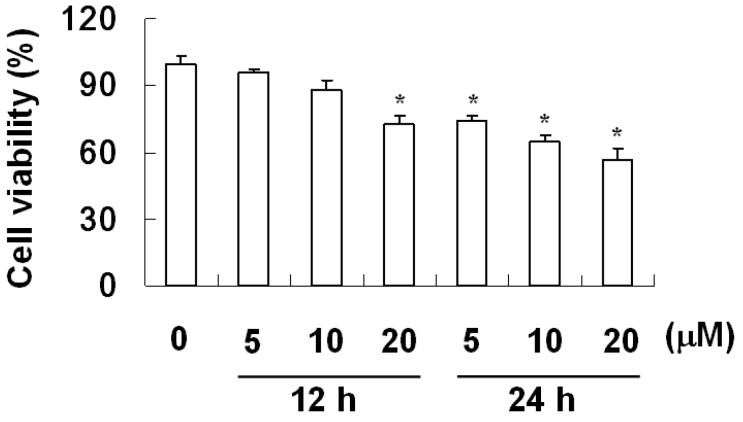
Representative cell viability of A549 cells treated by davallic acid at various concentrations for 12 h and 24 h, respectively. Results are expressed as mean ± SD (n = 3). * *p* < 0.05 was compared with the 0 µM concentration.

The effect of davallic acid in inhibiting A549 cell viability is shown in Figure 2. The results indicate that davallic acid (5, 10, and 20 μM) decreased A549 cell viability within 12 and 24 h of treatment to 73% and 57% with 20 μM davallic acid, respectively. These results suggest that davallic acid exhibits potential apoptotic induction, thereby repressing cell proliferation. Moreover, we evaluated the apoptosis index of A549 cells treated with davallic acid.

### 2.3. The Effect of Davallic Acid on Cell Cycle

**Figure 3:**
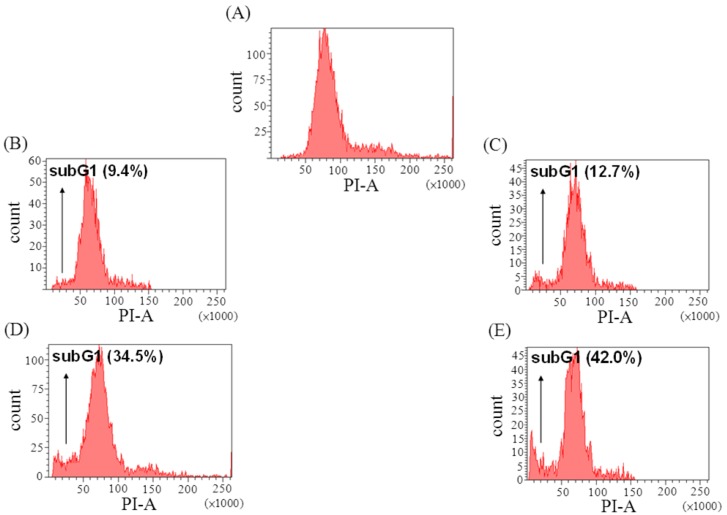
Effects of davallic acid on A549 apoptosis (sub G1 peak) after treatment for 12 h and 24 h, respectively. (**A**) control; (**B**) 10 μM treatment for 12 h; (**C**) 20 μM treatment for 12 h; (**D**) 10 μM treatment for 24 h; (**E**) and 20 μM treatment for 24 h.

The sub-G_1_ phase, in which DNA fragmentation peaks, represents apoptosis generation [12,13,14]. The sub-G_1_ phase was significantly increased by 34% and 42% following treatment with 10 and 20 μM davallic acid for 24 h, respectively, as compared to the control group (Figure 3).

**Figure 4:**
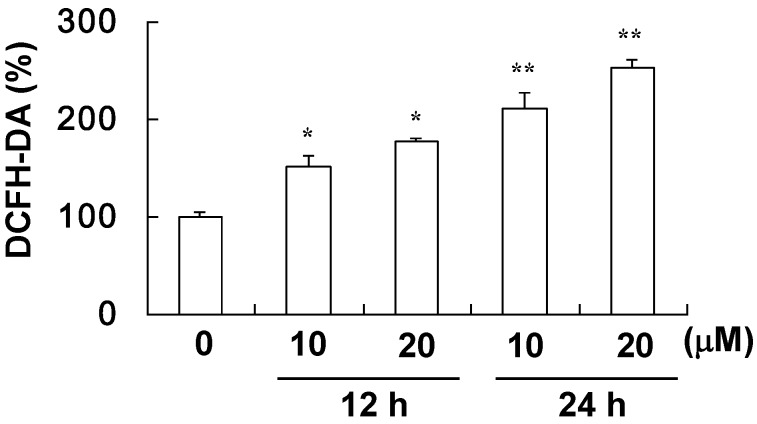
ROS production of A549 cells treating with davallic acid at various concentrations for 12 h and 24 h, respectively. Results are expressed as mean ± SD (n = 3). * *p* < 0.05 and ** *p* < 0.01 were compared with the first line that 0 µM concentration.

Cells were stained with DCFH-DA to examine the production of ROS in the A549 cells, and DCF fluorescence was measured by a flow cytometer. As shown in Figure 4, both 10 and 20 μM davallic acid significantly increased ROS levels after 12 and 24 h of treatment as compared to the control group. These results revealed that davallic acid induced apoptosis and suppressed cell viability by promoting the ROS levels, activating apoptotic signals in A549 cells. Therefore, we further investigated the apoptotic pathway induced by davallic acid.

### 2.4. The Effect of Davallic Acid on Bax and Bcl-2 Proteins in A549 Cell

**Figure 5:**
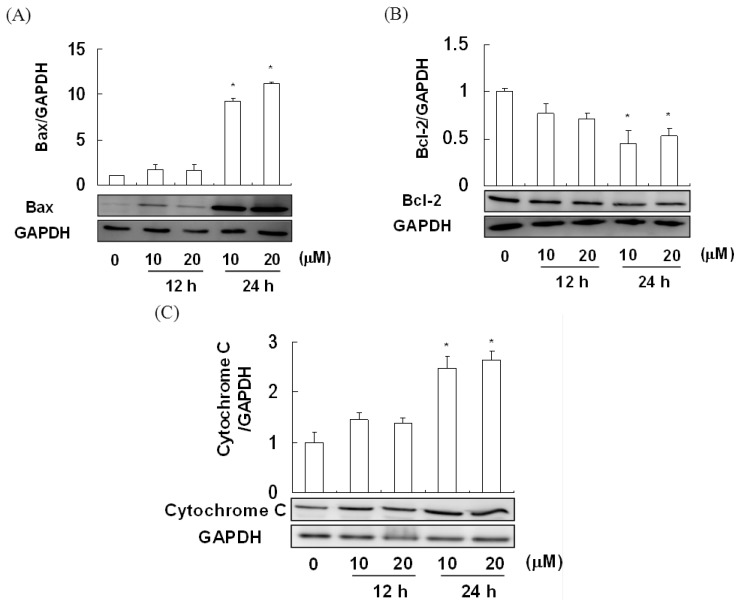
Bax and Bcl-2 expression of A549 cells treating with davallic acid at various concentrations for 12 h and 24 h, respectively. (**A**) Bax expression; (**B**) Bcl-2 expression; and (**C**) Cytosolic cytochrome C. Results are expressed as mean ± SD (n = 3). * *p <* 0.05 was compared with the first line that 0 µM concentration.

We observed that 10 and 20 μM davallic acid significantly increased cytosolic Bax levels in A549 cells treated for 24 h (Figure 5A). Furthermore, these treatments potentially suppressed a decrease in cytosolic Bcl-2 in A549 cells (Figure 5B). Moreover, we noted a significant increase in cytosolic cytochrome *c* in A549 cells treated with davallic acid (10 and 20 μM) for 24 h as compared to the control group (Figure 5C). These results suggest that davallic acid induced mitochondrial membrane damage and membrane potential loss, enhancing Bax expression and inhibiting Bcl-2 expression, subsequently leading to cytochrome *c* release.

### 2.5. The Effect of Davallic Acid on Caspase Activity

**Figure 6:**
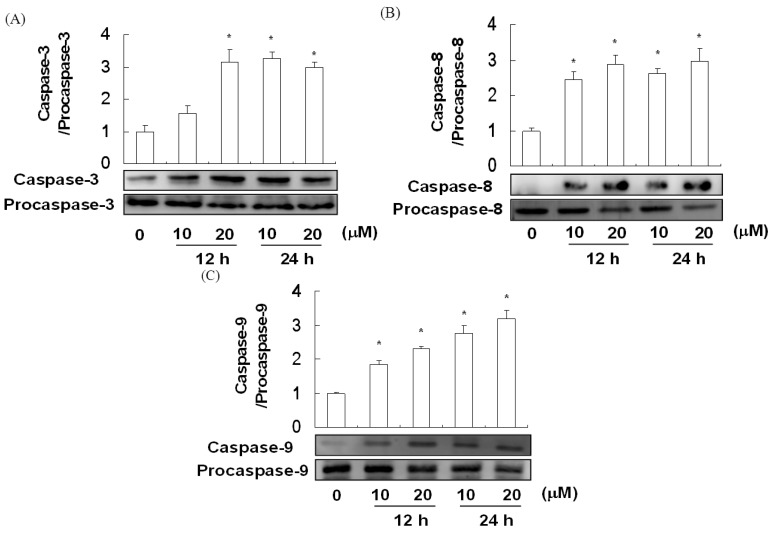
Caspases expression of A549 cells treating with davallic acid at various concentrations for 12 h and 24 h, respectively. (**A**) Caspase-3; (**B**) caspase-8; (**C**) and caspase-9. Results are expressed as mean ± SD (n = 3). * *p* < 0.05 was compared with the first line that 0 µM concentration.

Apoptosis is regulated by two main pathways: The TNF-α receptor pathway and the mitochondrial pathway, both of which involve the activation of caspase-3. Caspase-8 and caspase-9 are activators/initiators of the TNF-α receptor and mitochondrial pathways, respectively. The activities of caspase-3, caspase-8, and caspase-9 in A549 cells treated with davallic acid are shown in Figure 6A–C, respectively. Davallic acid (10 and 20 μM) treatment for 24 h significantly promoted the activation of caspase-3, caspase-8, and caspase-9 in the A549 cells as compared to that in the control group.

## 3. Discussion

Several lines of evidence demonstrate that excessive oxidative stress caused by ROS and reactive nitrogen species (RNS) can promote disease progression via the oxidation of biomolecules such as DNA, lipids, and proteins [15,16]. High levels of ROS in the mitochondria can result in free radicals attacking membrane phospholipids, which precedes mitochondrial membrane depolarization. Mitochondrial depolarization, which is considered an irreversible step in apoptosis, triggers a cascade of caspases [17,18].

The Bcl-2 family of proteins includes proteins that can either inhibit (e.g., Bcl-2, and Bcl-xL) or induce (e.g., Bax, Bak, and Bad) apoptosis. Bcl-2 is an antiapoptotic protein that is predominantly present in mitochondria and prevents apoptosis by suppressing oxyradical-mediated membrane damage and stabilizing mitochondrial membrane potential [19,20]. The proapoptotic protein Bax regulates the mitochondrial pathway by triggering the release of apoptotic factors such as cytochrome *c* from mitochondria following apoptotic signals [21]. Bcl-2 is known as an upstream effector molecule in the apoptotic pathway, and has been identified as a potent suppressor of apoptosis [22,23].

In addition, Bcl-2 promotes cell survival by preserving the integrity of the external mitochondrial membrane, which prevents the release of cytochrome *c* from mitochondria, thus preventing cell death [20,24]. Bax is a 21-kDa protein that promotes mitochondrial membrane permeability; it has been demonstrated to accelerate apoptotic cell death [25,26].

In recent years, natural food products have received increasing attention because of their potential roles in the prevention and/or intervention of cancers, including lung cancer. Oxidative damage to cellular macromolecules can arise through the overproduction of ROS and faulty antioxidant and/or DNA repair mechanisms that result in cancer [27]. Phytochemicals offer significant protective benefits against oxidative damage [14]. The antiproliferative activities of polyphenols such as delphinidin, cyanidin, peonidin, petunidin, and malvidin have been previously reported [28]. Davallic acid, as shown in the present study, suppressed cell viability and induced apoptosis in the A549 cancer cells via increased ROS expression, enhanced release of cytochrome *c*, and induction of caspase-3, caspase-8, and caspase-9 expression.

Six species of botanical rhizoma derivatives of Gusuibu have been found in Taiwan: *Drynaria fortune* (Kunze) J. Smith, *Pseudodrynaria coronans* (Wallich) Ching (both from Polypodiaceae), *Davallia divaricata* Blume, *Davallia mariesii* Moore ex Bak, *Davallia solida* (Forst) Swartz, and *Humata griffithiana* (Hooker) [2]. These six species have found uses in modern and traditional medicinal practices, including treatments for body discomfort, inflammation, cancer, ageing, blood stasis, and bone injuries [2]. Davallic acid is the major active compound of Gusuibu. Although the molecular structure of davallic acid was determined in 1965 [6], the bioactivity is yet be reported to date. A few studies have suggested that Gusuibu extract has cancer preventative properties [6,29]. While other studies have found that Gusuibu extract may inhibit A549 lung cancer cells invasion and migration. The study also reports that Gusuibu extract may restrain the tumor growth and migration *in vivo* [30]. Therefore, the davallic acid derivatives of Gusuibu may have applications as preventative agents to fight cancer which would indicate this compound merits further studies, and we feel the necessity to study the echanism of davallic acid bioactivity to determine its effectiveness.

## 4. Experimental

### 4.1. General

^1^H- and ^13^C NMR spectra were obtained on a Bruker AM-500 spectrometer in CDC1_3_ solution, using the corresponding solvent peak as the internal standard. Optical rotation was measured on a Jasco DIP-1020 digital polarimeter. Column chromatography was carried out with Sephadex LH-20 (25–100 µM, Pharmacia Fine Chemicals). TLC was conducted on silica gel plates (60 F-254, Merck) and 10% sulfuric acid solution was used as a visualizing agent following heating. Propidium iodide (PI), sodium dodecyl sulfate (SDS), Triton X-100, and trypsin were purchased from Sigma Chemical Co. (St. Louis, MO, USA). Fetal bovine serum (FBS) was purchased from Life Technologies (Auckland, New Zealand). Dimethyl sulfoxide (DMSO) was purchased from Wako Pure Chemical Industries (Saitama, Japan). Ham’s F-12, sodium bicarbonate, hydrocortisone, penicillin, and streptomycin were purchased from HyClone Laboratories (Logan, UT, USA). 

### 4.2. Plant Material and Isolation

The plant, *D. divaricata* Blume, collected in Taipei, Taiwan, in July 2010 was kindly supplied for this study by The Forestry Bureau of Taiwan. The sliced rhizomes were used for the extraction of davallic acid. The rhizomes (25 kg) were extracted with 80% EtOH (10 L) three times at room temperature. After evaporating the solvents under vacuum at 40 °C, a residue was obtained. This residue was dissolved in H_2_O and then extracted successively with hexane, ethyl acetate, and *n*-BuOH. The hexane extract (62 g) was fractionated on a Sephadex LH-20 column and eluted with EtOH to yield fractions A-D. The fraction C (5 g) was recrystallized from hexane/acetone. Finally, the sediment was washed with EtOH to give 1.5 g of davallic acid. The structure of the isolated compound was elucidated using Bruker Avance DRX 500 NMR and Agilent 1200/6460 LC-MS-MS for mass spectra confirmation. 

### 4.3. Cell Culture

A549 lung cancer cells were obtained from the Bioresource Collection and Research Center (BCRC) in Taiwan (Hsinchu, Taiwan), and cultured on F12-K medium supplemented with 10% heat-inactivated FBS and antibiotics (100 unit mL^−1^ penicillin and 100 μg mL^−1^ streptomycin). Cells were cultured at 37 °C in a humidified atmosphere of 5% CO_2_. 

### 4.4. Cell Viability

A549 cells were seeded into 48-well plates in 1 mL of F-12K medium. The cells were treated with different concentrations of samples. At the end of incubation, the cells were washed with PBS, and the supernatants were exchanged with 1 mL of medium and MTT (0.5 mg mL^−1^) to react for 2 h at 37 °C. After washing with PBS, MTT tetrazolium was dissolved with DMSO and measured at 570 nm [10].

### 4.5. Assay for ROS Level

A549 cells (2 × 10^6^ cells/per well) were treated with various concentrations of davallic acid for different durations of time (12 h and 24 h). The collected cells were suspended in 500 µL of PBS, mixed with 10 µM 2′,7′-dichlorfluorescein-diacetate (DCFH-DA) and incubated for 20 min at 37 °C. Afterwards, the cells were washed thrice to remove DCFH-DA. The cell pellet was then mixed with 500 µL of PBS and the ROS level was assayed by flow cytometry.

### 4.6. Cell Cycle Distribution

After exposure to davallic acid, the medium was aspirated and adherent cells were harvested and centrifuged at 300 × g for 5 min. Cells were washed with PBS, fixed with ice-cold ethanol at −20 °C overnight and then stained with PI at room temperature for 30 min. The cell cycle distribution was analyzed by flow cytometry using a FACScan-LSR flow cytometer equipped with CellQuest software (BD Biosciences, San Jose, CA, USA) [10].

### 4.7. Immunoblot Analysis

A549 cells were lysed in ice-cold lysis buffer containing 20 mM of Tris-HCl (pH 7.4), 1% of Triton X-100, 0.1% of SDS, 2 mM of EDTA, 10 mM of NaF, 1 mM of phenylmethylsulfonyl fluoride (PMSF), 500 μM of sodium-vanadate, and 10 μg/mL of aprotinin overnight. Then, cell lysates were sonicated with ice cooling (four times each 15 s) and centrifuged (12,000 × g, 10 min) to recover the supernatant. The supernatant was taken as the cell extract. The protein concentration in the cell extract was determined using a Bio-Rad protein assay kit. The samples were subjected to 10% sodium dodecyl sulfate-polyacrylamide gel electrophoresis (SDS-PAGE). The protein spots were electrotransferred to a polyvinylidene difluoride (PVDF) membrane. The membrane was incubated with a block buffer for 1 h, washed with PBS containing 0.05% Tween-20 (PBST) three times, and then probed with 1:5,000 diluted solution of anti-caspase-3, anti-procaspase-3, anti-caspase-8, anti-procaspase-8, anti-caspase-9, anti-procaspase-9, 1:2,000 diluted solution of anti-cytochrome, anti-Bcl-2, anti-Bax, and anti-GAPDH (1:5,000) (Cell Signaling Technology, Beverly, MA, USA) overnight at 4 °C. The membrane was washed three times each for 5 min in PBST, shaken in a solution of HRP-linked anti-rabbit IgG secondary antibody, and washed three more times each for 5 min in PBST. The expressions of proteins were detected using enhanced chemiluminescent (ECL) reagent (Millipore, Billerica, MA, USA).

### 4.8. Statistical Analysis

Data were expressed as mean ± standard deviation (SD). Statistical significance was determined by one-way analysis of variance (ANOVA) using the general linear model procedure of SPSS Version 17.0. [31], followed by ANOVA with Duncan’s test. All comparisons were performed relative to controls, and significant differences are indicated.

## 5. Conclusions

In conclusion, in this study we affirmed that davallic acid obtained from *D. divaricata* Blume induced cell death in lung A549 cancer cells, playing an important role in apoptosis induction via caspase-3, caspase-8, and caspase-9 activation. We therefore consider davallic acid may have potential as a natural anti-lung cancer compound, warranting further study.
